# Bulk RNA-seq and scRNA-seq reveal SLC7A11, a key regulatory molecule of ferroptosis, is a prognostic-related biomarker and highly related to the immune system in lung adenocarcinoma

**DOI:** 10.1097/MD.0000000000034876

**Published:** 2023-09-15

**Authors:** Xiaoyu Wu, Sheng Wang, Kaifang Chen

**Affiliations:** a Department of Respiratory, Zhejiang Jinhua Guangfu Cancer Hospital, Jinhua, Zhejiang, China; b Department of Emergency, Affiliated Jinhua Hospital, Zhejiang University School of Medicine, Jinhua, Zhejiang, China.

**Keywords:** ferroptosis, immune, lung adenocarcinoma, prognosis, SLC7A11

## Abstract

Lung adenocarcinoma (LUAD) is the most common pathological subtype of lung cancer. Ferroptosis is an iron-dependent, non-apoptotic cell death mode, highly correlated with the tumorigenesis and progression of multiple cancers. Solute carrier family 7 member 11 (SLC7A11) maintains the anti-porter activity of cysteine and glutamate to regulate ferroptosis. We collected bulk RNA-seq and scRNA-seq from The Cancer Genome Altas and Gene Expression Omnibus databases. Then, we extracted the expression level of SLC7A11 to perform the differential expression analysis between normal tissues and LUAD tissues. Then, we applied survival, univariate, and multivariate Cox regression analyses to investigate the predictive value of SLC7A11 in LUAD. Gene set enrichment analysis was used to explore the underlying molecular mechanisms of SLC7A11 in LUAD. Finally, we analyzed the relationship of SLC7A11 to the immune status and the curative effect of immunotherapy. The expression level of SLC7A11 in LUAD tissues was markedly increased. The survival analysis, univariate and multivariate Cox regression analysis showed that SLC7A11 was a negative factor for the prognosis of LUAD patients. Gene set enrichment analysis revealed that several immune-related pathways were enriched in the low-level group. The lower SLC7A11 level has a better therapeutic effect of immunotherapy and less probability of immune escape and dysfunction. SLC7A11 was a prognostic-related biomarker and closely correlated with the immune status and therapeutic effect of immunotherapy in LUAD, which could be an effective biomarker for evaluating the prognosis and the therapeutic efficacy of immunotherapy.

## 1. Introduction

For both sexes, lung cancer (LC) is one of the most commonly diagnosed malignancies and the leading cause of cancer-related mortality globally.^[1,2]^ It was estimated that more than 220,000 new cases were diagnosed with LC in 2020, and over 180,000 patients died of LC.^[3,4]^ According to histopathological classification, LC could be divided into non-small cell lung cancer (NSCLC) and small cell lung cancer.^[2]^ NSCLC is the most common subtype of LC and accounts for approximately 85% of LC.^[3]^ About 65% of cases are lung adenocarcinoma (LUAD).^[3]^ In recent years, new diagnostic techniques and treatment strategies have been emerging and prominently prolonged the survival time of LUAD patients. However, the 5-year survival rate of LUAD patients is still <20%.^[4,5]^ Therefore, it is essential to identify tumor-specific predictive biomarkers and to understand the molecular mechanisms underlying LUAD, which may be conducive to risk assessment and guide clinical decision-making of LUAD.

In 2012, Professor Dixon proposed the theory of ferroptosis for the first time.^[6]^ Ferroptosis is an iron-dependent, non-apoptotic cell death mode.^[6,7]^ Distinct from the traditional cell death model: apoptosis and autophagy, ferroptosis is specifically characterized by the aggregation of lipid reactive oxygen species (ROS) and intracellular iron and changes of cytological morphology, such as the rupture of the outer mitochondrial membrane, reduction of mitochondrial membrane, and vanishing of mitochondria cristae.^[8–10]^ Glutathione and ROS were the main regulating modes of ferroptosis.^[9,11]^ In the past, apoptosis and autophagy were considered to be the main pathways leading to cancer cell death. Nevertheless, increasing studies have found that excessive ROS accumulation will contribute to irreversible oxidative damage and inhibit tumor cell proliferation. Furthermore, ferroptosis could significantly enhance the efficacy of killing tumor cells, suggesting that ferroptosis may be a novel cancer treatment strategy.^[12]^ In addition to cancer cell death, ferroptosis may play an important role in stroke, neurogenerative diseases, cardiomyopathy, and even traumatic brain injury.^[13–16]^

According to the regulation of neutral reaction to ROS, ferroptosis inducers can be divided into 2 classes: Class I and II. Class I ferroptosis inducers function via inhibiting GPX4, such as RSL3, ML162, and FIN56. Class II ferroptosis inducers, such as erastin, sorafenib, and sulfasalazine, function by blocking system Xc−, a transporter located in the cell membrane and capable of decreasing the glutathione level. System Xc− is a sodium-independent anti-porter responsible for maintaining redox homeostasis by importing cystine into the cell and exporting glutamate out of the cell simultaneously.^[17,18]^ It structurally consists of 2 subunits: Solute carrier family 7 member 11 (SLC7A11) and Solute carrier family 3 member 2 (SLC3A2).^[19,20]^ SLC7A11, a multi-pass transmembrane protein, maintains the cystine/glutamate anti-porter activity,^[21]^ whereas SLC3A2 anchors SLC7A11 to the plasma membrane and maintains SLC7A11 protein stability. After being imported into the cell through SLC7A11, cystine is converted to cysteine via an NADPH-consuming reduction reaction. Subsequently, cysteine conjugates with glutamate to form γ-glutamylcysteine. Then, glutathione synthetase-mediated enzymatic addition of a glycine molecule works to produce glutathione. GPX4 uses glutathione to reduce lipid hydroperoxides to lipid alcohols to suppress ferroptosis.^[22,23]^ Many studies have reported that SLC7A11 overexpressed in several cancer cells and could promote glutathione biosynthesis and ferroptosis resistance.^[19]^ In NSCLC, studies have shown that SLC7A11 could regulate metabolic requirements to accelerate cancer progression and repress LC growth by inhibiting SLC7A11, hinting that SLC7A11 may be a potential therapeutic target for LC.^[24–26]^ Hence, in the current study, the bulk RNA data and Single-cell RNA sequencing (scRNA-seq) data were downloaded from The Cancer Genome Altas (TCGA, https://www.cancer.gov) and Gene Expression Omnibus (GEO, https://www.ncbi.nlm.nih.gov). Then, we explored the expression profile of SLC7A11 in LUAD and found that the expression level of SLC7A11 was significantly higher in LUAD tissues. Moreover, the study showed that SLC7A11 was highly related to the prognosis and immune status of LUAD patients.

## 2. Materials and Methods

### 2.1. Data collection

The study downloaded 3 independent datasets (TCGA-LUAD, GSE68465, and GSE31210) from 2 public functional genomics databases: TCGA and GEO. The Bulk RNA profile for the TCGA database was level 3 RNA sequencing. The gene expression profiles in the GSE68465 and GSE31210 databases were raw data and normalized with the robust multi-array average algorithm via the R package Affy (3.17). Both the platform of GSE68465 and GSE31210 were GPL96 (Affymetrix Human Genome U133A Array). In addition, the corresponding clinical data of all participants were also collected, including age, gender, TNM stage, overall survival time (OS), and survival status. A total of 1122 patients with LUAD were enrolled in the study, including 478 from the TCGA dataset, 418 from the GSE68465 dataset, and 226 from the GSE31210 dataset. All patient’s detailed demographic and baseline information was presented in Table 1. The scRNA-seq data (including 2 normal lung tissues and 4 LUAD tissues) was gathered from the GEO database (GSE149655, GSE171145). The patients involved in the database have obtained ethical approval. Our study is based on open-source data, so there are no ethical issues or other conflicts of interest.

**Table 1:** The baseline characteristics of lung adenocarcinoma patients in this study.

Parameter	TCGA-LUAD	GSE68465	GSE31210
Gender			
Female	260 (54.39%)	215 (51.44%)	121 (53.54%)
Male	218 (45.61%)	203 (48.56%)	105 (46.46%)
Age			
≤65	239 (50.00%)	218 (52.15%)	176 (77.88%)
>65	239 (50.00%)	200 (47.85%)	50 (22.12%)
TNM stage			
I/II	378 (79.08%)	363 (86.84%)	226 (100%)
III/IV	100 (20.92%)	55 (13.16%)	0
Tumor size			
T1–2	417 (87.24%)	381 (91.15%)	NA
T3–4	61 (112.76%)	37 (8.85%)	NA
NA	0	0	226 (100%)
Lymph node			
N0	313 (65.48%)	289 (69.14%)	NA
N1–3	165 (34.52%)	129 (30.86%)	NA
NA	0	0	226 (100%)
Metastasis			
M0	453 (94.77%)	418 (100%)	NA
M1	23 (5.23%)	0	NA
NA	0	0	226 (100%)
Survival status			
Alive	309 (64.64%)	197 (47.13%)	191 (84.51%)
Dead	169 (35.36%)	221 (52.87%)	35 (15.49%)
SLC7A11			
Low	239 (50.00%)	209 (50.00%)	113 (50.00%)
High	239 (50.00%)	209 (50.00%)	113 (50.00%)
Total	478 (100%)	418 (100.00%)	226 (100%)

GEO = Gene Expression Omnibus; NA = represents information not available; TCGA = The Cancer Genome Altas.

### 2.2. Differential expression analysis of SLC7A11

The differential mRNA expression level of SLC7A11 between normal and LUAD tissues was compared with the Wilcoxon rank-sum test. Also, the Wilcoxon rank-sum test explored the relationship between the expression level of SLC7A11 and the patient’s baseline characteristics. *P*-value < 0.05 was deemed to be a statistical difference. The expression level of SLC7A11 in Pan-cancer was investigated with The TIMER (Version: 2, http://timer.comp-genomics.org).^[27]^

### 2.3. Protein–protein interaction network

With confidence >0.7 as the threshold, the protein–protein interaction network of SLC7A11 was gathered from String (Version: 11.5, https://www.string-db.org) to explore the proteins interacting with SLC7A11.^[28]^

### 2.4. ScRNA-seq data processing

The R package: “Seurat” was used to analyze the transcript count matrix for quality control and preliminary data exploration. The filtering threshold was set as follows:

Excluding genes detected in <3 cells.

Excluding cells with <50 genes detected.

Excluding cells with >10% mitochondrial gene expression.

The expression profiles were then normalized with the Log Normalization algorithm and subsequently normalized using a linear regression model. The top 2000 highly expressed and variable genes were selected for principal component analysis to determine significant and influential dimensions. The t-Stochastic Neighbor Embedding algorithm was used to reduce the dimension of the top 20 principal components and gather major cell clusters. The marker genes between difference clusters were identified with |log2 (fold change) |>1 and adjusted *P* value < 0.05 as the threshold. Cell annotation was carried out with the SingleR package^[29]^ and reports from the literature.^[30–32]^ Finally, single-cell trajectory analysis was performed with the “Monocle 2 algorithm.”^[33]^

### 2.5. Construction of a prognostic nomogram

We divided patients into low- and high-expression groups based on the median value of the SLC7A11 expression. Using clinical characteristics (age, gender, TNM stage) and the expression level of SLC7A11, we develop a prognostic nomogram in TCGA set for predicting 1-, 3-, and 5-year OS of LUAD patients. In addition, the receiver operating characteristic curve and the calibration plot were depicted, and the area under the curve was calculated to assess the predictive power of the nomogram.

### 2.6. Gene set enrichment analysis (GSEA)

To determine the basic biological mechanisms of SLC7A11, we carried out the GSEA analysis. A false discovery rate (FDR) < 0.05 was set as the criteria.

### 2.7. Investigation of the association between SLC7A11 and the immune system and therapy

The immune- and stromal-scores in the tumor microenvironment (TME) were calculated with the R package: “ESTIMATE.” Furthermore, the single-sample gene set enrichment analysis was performed with the BiocManager package: GSVA to determine the level of tumor-infiltrating immune cells (TIICs) and the activity of immune-related functions. The Cancer Immunome Atlas (https://tcia.at/) provides an immunophenoscore (IPS) value, which was analyzed by analyzing the next-generation sequencing data of 20 solid tumors.^[34]^ The IPS value reflects the response to immune checkpoint inhibitor treatment. The higher IPS value represents a better response to immune checkpoint inhibitor treatment. The tumor immune dysfunction and exclusion (TIDE, http://tide.dfci.harvard.edu) is a public database for predicting tumor immune dysfunction and exclusion. The higher score represents a worse response to immune therapy. In addition, we estimated the relation of the SLC7A11 expression level to the susceptibility of 23 common antitumor drugs in LUAD (Afatinib, Alectinib, brigatinib, Cabozantinib, Carboplatin, Cisplatin, Crizotinib, Dacomitinib, Docetaxel, Erlotinib, Etoposide, Fluorouracil, Gefitinib, Gemcitabine, Homoharringtonine, Irinotecan, Osimertinib, Oxaliplatin, Paclitaxel, Pemetrexed, Vinblastine, Vincristine, Vinorelbine) using Cellminer (Version:2021.1, https://discover.nci.nih.gov/cellminer/home.do).^[35]^

### 2.8. Tumor mutational burden (TMB)

The DNA somatic mutation data of corresponding LUAD patients was also downloaded from TCGA and further analyzed with the “maftools” R package.

### 2.9. Statistical analysis

The categorical and measurement data were presented as the number and the mean ± standard deviation. The group comparison of measurement data was conducted with a Wilcoxon rank-sum test or one-way ANOVA. Correlation analysis was performed using Spearman correlation test. The Kaplan–Meier plot and log-rank test were used to evaluate the relation of OS time to the mRNA expression level of SLC7A11. Univariate and multivariate Cox regression model were implemented to validate the prognostic significance of SLC7A11. All statistical analyses were performed with R (Version: 4.1.1, https://www.r-project.org).

## 3. Results

### 3.1. Bulk RNA-seq revealed the expression level of SLC7A11 in LUAD

**Figure 1.**
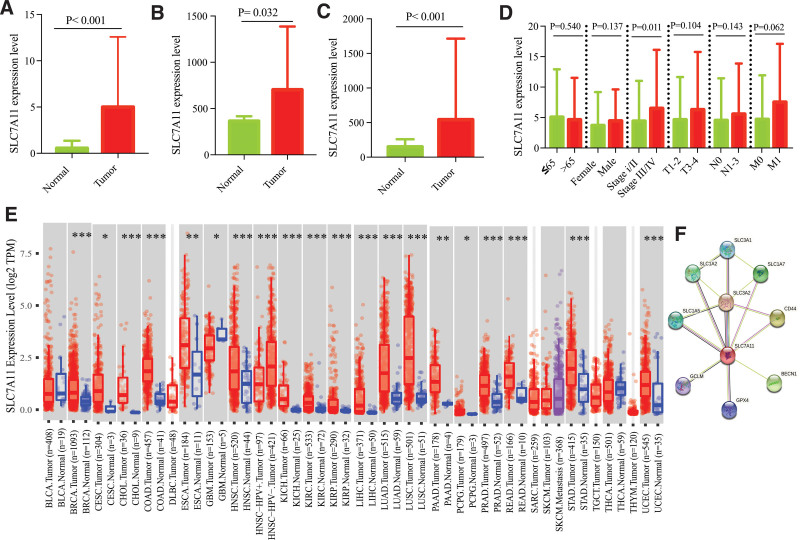
Bulk RNA-seq revealed the expression level of SLC7A11 in LUAD. (A) The SLC7A11 level was increased in LUAD in the TCGA dataset, (B) in GSE68465, (C) GSE31210. (D) The relation of SLC7A11 to clinical parameters. (E) The expression level of SLC7A11 in Pan-cancer. (F) A PPI network of SLC7A11. LUAD = lung adenocarcinoma; PPI = protein–protein interaction. *P < .05; **P < .01; ***P < .001.

As shown in Figure 1A–C, the SLC7A11 expression level in LUAD was significantly increased (TCGA: *P* < .001; GSE68465: *P* = .032; GSE31210: *P* < .001). Moreover, the LUAD patients staged at III/IV have a higher SLC7A11 expression level than the LUAD patients staged at I/II (*P* = .011, Fig. 1D). Pan-cancer analysis revealed that, compared with that in the normal tissues, the expression level of SLC7A11 was up-regulated in several types of cancers (Fig. 1E). Then, we investigated the proteins interacting with SLC7A11, using the confidence > 0.9 as the threshold. We found that 9 proteins were highly relevant to SLC7A11, of which multiple proteins played a vital role in cell ferroptosis (Fig. 1F).

### 3.2. ScRNA-seq revealed the expression level of SLC7A11 in LUAD

**Figure 2.**
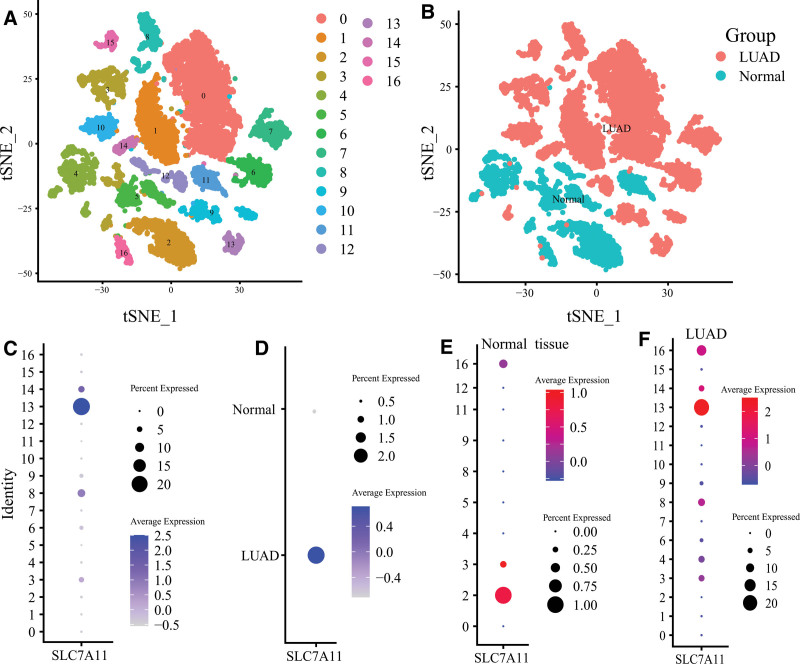
Cell clustering and the expression level of SLC7A11 in each cluster. (A) The t-SNE algorithm divided the cells into 17 clusters with 20 principal components. (B) The distribution of each cluster in normal and LUAD tissues. (C) The expression level of SLC7A11 in each cluster. (D) The expression level of SLC7A11 in normal and LUAD tissues. The SLC7A11 expression level of all clusters in (E) normal tissues and (F) LUAD tissues. t-SNE = t-Stochastic Neighbor Embedding.

Before filtering, we identified 5423 cells in 2 normal samples and 12,315 cells in 4 LUAD samples. After data standardization and quality control, 14,822 cells (4313 in normal samples and 10,509 in LUAD samples) were selected. The principal component analysis was performed to reduce data dimensionality reduction. Then, the top 2000 highly expressed and variable genes were picked out for further analysis. Nonlinear dimension reduction was carried out with the t-Stochastic Neighbor Embedding algorithm, which successfully clustered the cells into 17 clusters (Fig. 2A). The expression level of SLC7A11 was most abundant in Cluster 13 (Fig. 2C). In normal tissues, 10 clusters (Cluster 0, 2, 3, 4, 5, 8, 9, 11, 12, 16) were identified. In LUAD tissues, 15 clusters were identified (except Cluster 5). Between normal and LUAD tissues, cells were enriched in different clusters (Fig. 2B). In addition, the expression of SLC7A11 was significantly increased in LUAD tissues (Fig. 2D). Cluster with the highest SLC7A11 expression level in normal tissues was Cluster 2, whereas in LUAD tissues was Cluster 13 (Fig. 2E and F).

**Figure 3.**
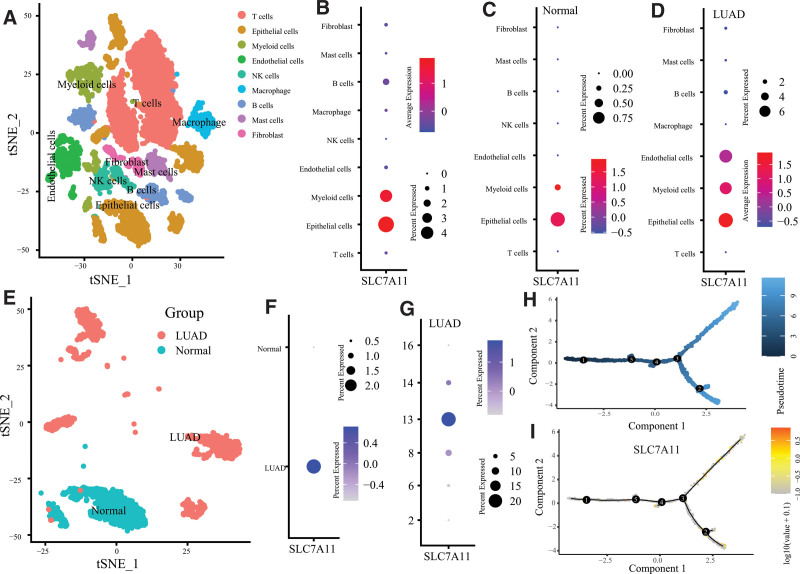
Cell annotation and the pseudo-time cell differentiation trajectory analysis. (A) The tSNE plot revealing 17 clusters were annotated into 9 different cell types. The SLC7A11 expression level of each cell type in (B) all samples, (C) normal samples, (D) LUAD samples. (E) The tSNE plot presented the distribution of epithelial cells in normal and LUAD samples. (F) The SLC7A11 expression level of epithelial cells in normal and LUAD samples. (G) The SLC7A11 expression level of each cluster of Epithelial cells in LUAD samples. (H) The trajectory analysis of Epithelial cells with top 10 marker genes. (I) The SLC7A11 expression level in different developmental states.

Then, we annotated all clusters into 9 cell types (Fig. 3A). The expression of SLC7A11 was highest in Epithelial cells (Fig. 3B). Similar results were observed in normal tissues (Fig. 3C) and LUAD tissues (Fig. 3D). In this study, 6 clusters (Cluster 2, 6, 8, 13, 14, 16) were annotated into epithelial cells, which mainly contained lung epithelial cells, cancer cells, and cancer stem cells. Epithelial cells were primarily enriched in Cluster 2 and 16 for normal tissues. In contrast, for LUAD tissues in Cluster 6, 8, 13, and 14 (Fig. 3E). Similarly, the SLC7A11 expression of epithelial cells in LUAD tissues was higher than that in normal tissues (Fig. 3F). The most decadent Cluster in LUAD tissues was Clusters 13 (Fig. 3G). We included the epithelial cells in the pseudo-time cell differentiation trajectory analysis. The results were presented in Figure 3H and demonstrated the evolutionary pattern of epithelial cells. Figure 3I showed the SLC7A11 expression in different developmental states.

### 3.3. Increased SLC7A11 level predicts poorer prognosis in LUAD

**Figure 4.**
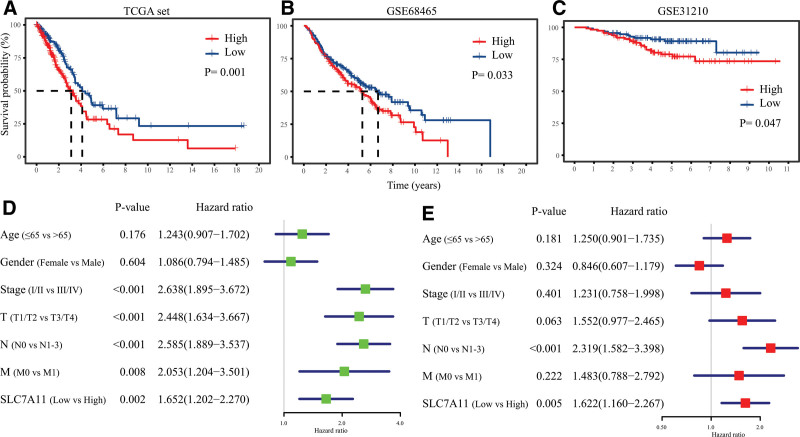
Increased SLC7A11 level predicts poorer prognosis in LUAD. Survival analysis showed SLC7A11 was an unfavorable factor for the prognosis of LUAD patients, (A) in the TCGA dataset, (B) in GSE68465, (C) GSE31210. (D) The univariate and (E) multivariate Cox regression analysis in the TCGA dataset revealed the SLC7A11 expression level was an independent prognostic factor for the prognosis of LUAD.

Based on the median value of the SLC7A11 expression, we divided patients into low- and high-expression groups. The Kaplan–Meier plot was carried out to evaluate the associations between survival differences between high- and low-expression groups. The survival analysis in the TCGA dataset demonstrated that the higher SLC7A11 expression level had poorer clinical outcomes (*P* = .001, Fig. 4A). Similar results were observed in the GSE68465 (*P* = .033, Fig. 4B) and GSE31210 (*P* = .047, Fig. 4C) datasets.

Then, we applied univariate and multivariate Cox regression analysis to compare the SLC7A11 expression level with clinical indexes in the 3 datasets to investigate whether the SLC7A11 expression level was an independent prognostic predictor in LUAD. The univariate Cox regression analysis revealed that the SLC7A11 expression level was a disadvantageous influence factor for the prognosis of LUAD patients (hazard ratio [HR] = 1.652, 95% confidence interval [CI] [1.202–2.270], *P* = .002, Fig. 4D). The finding was confirmed in the GSE68465 (HR = 1.434, 95% CI [1.098–1.874], *P* = .008, Fig. S1A, Supplemental Digital Content, http://links.lww.com/MD/J592) and GSE31210 (HR = 2.340, 95% CI [1.146–4.779], *P* = .020, Fig. S1C, Supplemental Digital Content, http://links.lww.com/MD/J592) datasets, in which similar results were obtained. Additionally, the multivariate Cox regression analysis in the TCGA dataset indicated that the SLC7A11 expression level was an independent prognostic factor for the OS time of LUAD (HR = 1.622, 95% CI [1.160–2.267], *P* = .005, Fig. 4E). We validated the results in the GSE68465 (HR = 1.351, 95% CI [1.023–1.785], *P* = .034, Fig. S1B, Supplemental Digital Content, http://links.lww.com/MD/J592) and GSE31210 (HR = 2.243, 95% CI [1.066–4.718], *P* = .033, Fig. S1D, Supplemental Digital Content, http://links.lww.com/MD/J592) datasets.

### 3.4. Construction of a prognostic nomogram

**Figure 5.**
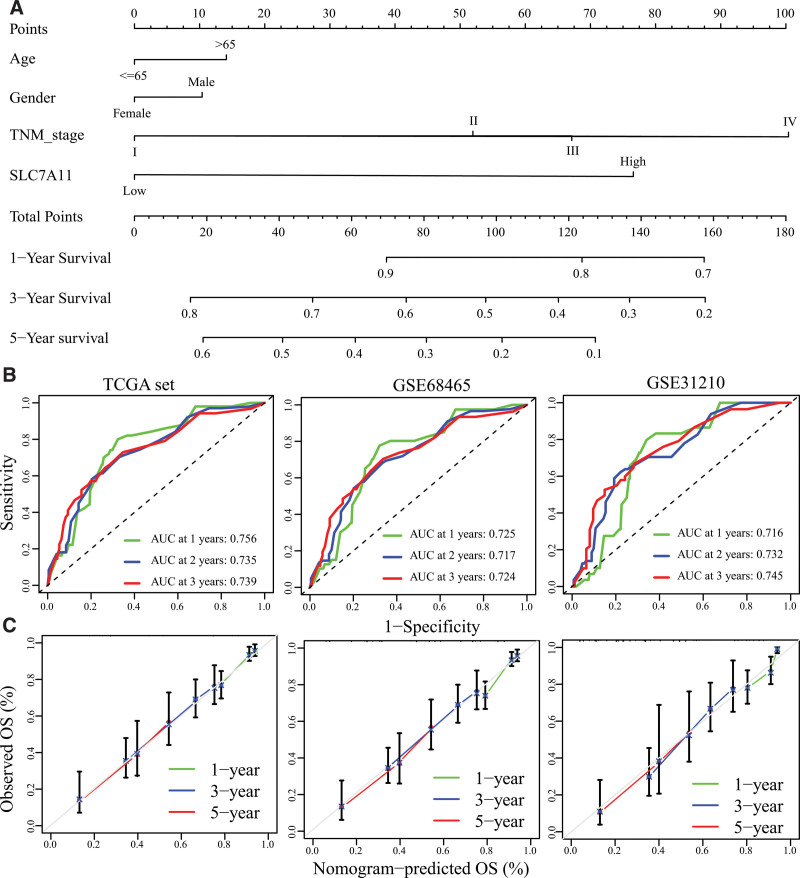
Construction of a prognostic nomogram. (A) The prognostic nomogram for predicting 1-, 3-and 5-year overall survival in LUAD. (B) The ROC of the nomogram. (C) The calibration plots of the nomogram. ROC: The receiver operating characteristic curve.

We took advantage of clinical characteristics (age, gender, TNM stage) and the expression level of SLC7A11 to construct a prognostic nomogram in the TCGA set (Fig. 5A). The area under the curves of the nomogram for predicting 1-, 3- and 5-year OS in TCGA set reached 0.756, 0.735, and 0.739, respectively (Fig. 5B). In GSE68465 were 0.725 (1-year), 0.717 (3-year) and 0.724 (5-year), and in GSE31210 were 0.716 (1-year), 0.732 (3-year) and 0.745 (5-year). In addition, the calibration plot showed an optimal fit with the ideal model (Fig. 5C)

### 3.5. GSEA analysis

**Figure 6.**
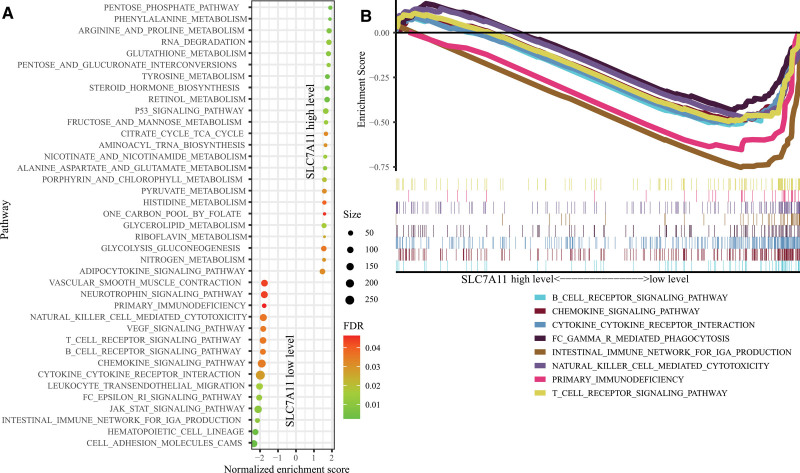
Gene set enrichment analysis (GSEA) analysis between low-and high-level SLC7A11 groups. (A) A total of 39 pathways were enriched, including 24 pathways in the high-level group and 15 pathways in the low-level group. (B) Immune-related pathways enriched in the low-level group.

To explore the potential altered signaling pathways between the low- and high-level SLC7A11 group, we conducted the GSEA analysis. The analysis found 39 enriched pathways (Fig. 6A), including 24 pathways in the high-level SLC7A11 group and 15 in the low-level SLC7A11 group. Of note, in the low-level SLC7A11 group, several immune-related pathways were identified (Fig. 6B), such as the T cell receptor signaling pathway (normalized enrichment score [NES] = −1.77, FDR = 0.035), B cell receptor signaling pathway (NES = −1.78, FDR = 0.035), Chemokine signaling pathway (NES = −1.82, FDR = 0.034), cytokine–cytokine receptor interaction (NES = −1.89, FDR = 0.027), Fc gamma R signaling pathway (NES = −1.99, FDR = 0.013), intestinal immune network for IgA production (NES = −2.09, FDR = 0.011), primary immunodeficiency (NES = −1.72, FDR = 0.042), and natural killer cell-mediated cytotoxicity (NES = −1.75, FDR = 0.035).

### 3.6. SLC7A11 is highly associated with immune status

**Figure 7.**
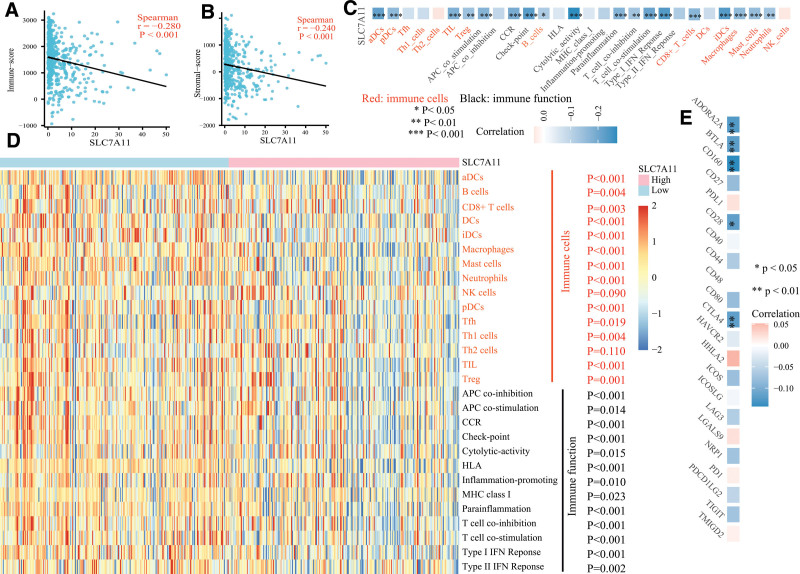
SLC7A11 is highly associated with immune status. The SLC7A11 expression level was negatively related to (A) the immune-score and (B) the stromal-score, (C) several immune cells and immune functions. (D) The distribution of the enrichment scores of immune cells and immune functions between low- and high-level SLC7A11 group. (E) The relationship of SLC7A11 to immunomodulators. aDCs = activated dendritic cells; DCs = dendritic cells; iDCs = immature dendritic cells; pDCs = plasmacytoid dendritic cells; Tfh = T follicular helper cells; Th1 cells = type 1 helper cells; Th2 cells = type 2 helper cells; TIL = tumor infiltrating lymphocytes; Treg: = regulatory T cells; HLA = human leukocyte antigen; CCR = C–C chemokine receptor; APC = antigen presenting cells; MHC = major histocompatibility complex. *P < .05; **P < .01; ***P < .001.

From GSEA analysis, we found that the different expression levels of SLC7A11 altered the enrichment of several immune-related pathways. Therefore, further analysis assessed the association between the SLC7A11 expression level and immune status. As shown in Figure 7A and B, the SLC7A11 expression level was negatively associated with the immune-score (*P* < .001) and stromal-score (*P* < .001). Then, we determined the association between the SLC7A11 expression level, TIICs, and immune functions. Interestingly, in the low-level SLC7A11 group, the enrichment scores of most immune cells and functions were enhanced (Fig. 7D). Moreover, the Spearman correlation test revealed that the enrichment scores of 10 immune cells (aDCs: *P* < .001; pDCs: *P* < .001; iDCs: *P* < .001; TIL: *P* < .001; Treg: *P* = .002; B cells: *P* = .010; CD8 + T cells: *P* < .001; macrophages: *P* < .001; mast cells: *P* < .001; neutrophils: *P* = .004) and 8 immune functions (APC co-inhibition: *P* < .001; CCR: *P* < .001; check-point: *P* < .001; cytolytic-activity: *P* < .001; parainflammation: *P* < .001; T cell co-inhibition: *P* = .003; T cell co-stimulation: *P* < .001; Type I IFN reponse: *P* < .001) were negatively associated with the SLC7A11 expression level (Fig. 7C). In addition, the expression levels of several immunomodulators were also negatively related with the SLC7A11 expression level (ADORA2A: *P* = .005; BTLA: *P* = .008; CD160: *P* = .002; CTLA4: *P* = .009, Fig. 7E).

### 3.7. SLC7A11 is highly associated with immunotherapy

**Figure 8.**
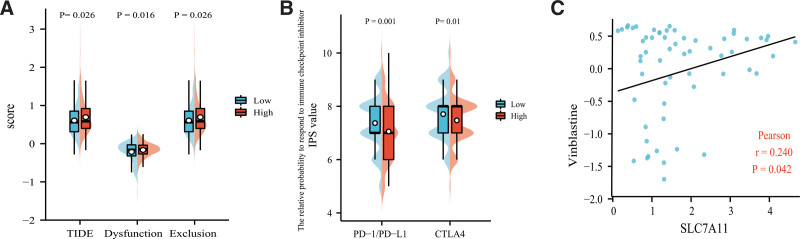
SLC7A11 is highly associated with immunotherapy. (A) The lower SLC7A11 level had less probability of immune escape and immune dysfunction, (B) and the better curative effect of anti-PD-1/PD-L1 and anti-CTLA4 treatment. (C) The SLC7A11 expression level was only positively related to the susceptibility of vinblastine.

We analyzed the scores of TIDE, dysfunction, and exclusion, which reflected the potential of immune evasion and the curative effect of immunotherapy. As shown in Figure 8A, patients with higher SLC7A11 expression level had higher TIDE (*P* = .026), dysfunction (*P* = .016), and exclusion score (*P* = .026), indicating that patients with low SLC7A11 level were more likely to benefit from immunotherapy. The IPS values exhibited similar results that the IPS values of anti-PD-1/PD-L1 (*P* = .010) and anti-CTLA4 (*P* = .001) treatment in patients with high SLC7A11 level were descending (Fig. 8B). Next, we assessed the association between the SLC7A11 expression level and the susceptibility of 23 common antitumor drugs in LUAD. It was found that the SLC7A11 expression level was only positively related to the vulnerability of Vinblastine (*P* = .042, Fig. 8C).

### 3.8. Tumor mutational burden

**Figure 9.**
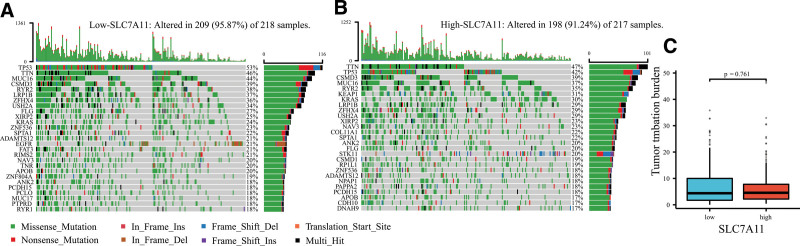
The TMB characteristics. (A) Mutational landscape in the low-SLC7A11 patients. (B) Mutational landscape in the high-SLC7A11 patients. (C) The difference in TMB between the 2 groups. TMB = tumor mutational burden.

The mutational landscape showed that the frequent mutation events in the low-SLC7A11 patients (95.87%) were more than those in the high-SLC7A11 patients (91.24%) (Fig. 9A and B). In the low-SLC7A11 patients, the most frequently mutated gene was TP53 (53%), followed by TTN (46%) and MUC56 (44%). In the high-SLC7A11 patients, the top 3 frequently mutated genes were TTN (47%), TP53 (42%), and CSMD3 (39%). However, there was no difference in TMB between the low- and high-SLC7A11 patients (*P* = .761, Fig. 9C).

## 4. Discussion

Ferroptosis is an additional proposed regulated cell death, which induces cell injury or death via the iron-dependent lipid ROS aggregation. Pathophysiological characteristics of ferroptosis distinguish it from cell necrosis and apoptosis.^[36]^ Emerging studies have revealed that the induction of ferroptosis is involved in various cancers’ pathological progressions and growth. Ferroptosis could be a preventive or therapeutic target to promote tumor cell death.^[37]^

In the System Xc-, SLC7A11 and SLC3A2 are linked by a disulfide bond and co-mediate the transport of cystine and glutamate, which are the main compositions of glutathione, the most abundant cellular antioxidant.^[17]^ It has been reported that SLC7A11 participated in the progression and development of many malignancies, including LC. For example, a study reported that suppressing the PRIM2/SLC7A11 axis could repress LC cells’ proliferation and colony formation.^[26]^ Another study found that in KRAS-mutant LUAD, inhibiting the SLC7A11/glutathione axis contributed to the synthetic lethality, which offered a potential therapeutic method for incurable KRAS-mutant LUAD.^[25]^ In addition, Ji et al demonstrated that SLC7A11 could regulate metabolic requirements during LC progression, and reducing SLC7A11 could control the development of LC.^[25]^ In the study, we identified that the SLC7A11 expression level in LUAD tissues was up-regulated, which was in line with previous studies.^[38,39]^ Moreover, in patients with stage III/IV, the SLC7A11 expression level was also increased. Nevertheless, until now, no study has reported the association between SLC7A11 expression level and the prognosis of LUAD. Thus, it was investigated in the current study. The survival analysis showed that the high expression level of SLC7A11 predicted a poorer prognosis of LUAD. Furthermore, a univariate and multivariate Cox regression analysis indicated that the SLC7A11 expression level was an independent prognostic predictor in LUAD. These results demonstrated that SLC7A11 was a prognostic-related biomarker for LUAD, and SLC7A11 may be a potential therapeutic target for LUAD.

To investigate the function of SLC7A11 and its mechanism of action in LUAD, we carried out the GSEA analysis. The results revealed that 39 pathways were determined between the high- and low-level SLC7A11 group. Among them, several pathways, which were highly related to the tumorigenesis and development of cancers, were enriched, such as the P53 signaling pathway, Jak-stat signaling pathway, etc. In addition, in the low-level SLC7A11 group, multiple immune-related pathways were identified, which may be the reason for the better prognosis in patients with low SLC7A11 expression level. Previous studies have reported that cancer cells undergoing ferroptosis could influence anti-tumor immunity by producing excessively oxidized lipid mediators. Furthermore, the induction of ferroptosis enhanced tumor growth by suppressing the immune system and affected the therapeutic effect of immunotherapy. It suggested that ferroptosis was highly associated with the immune system, which may function, in part, by regulating the immune system.^[40–42]^ Research on hepatocellular carcinoma,^[11]^ ovarian carcinoma,^[43]^ clear cell renal cell carcinoma,^[44]^ and adrenocortical carcinoma^[45]^ showed that SLC7A11 expression level was closely associated with the TIICs and immune functions. However, no study reported the relationship of SLC7A11 to the immune system in LUAD. Therefore, we estimated the immune-score and stromal-score in TME with the ESTIMATE algorithm. It was found that the immune-score and stromal-score were negatively correlated with the expression level of SLC7A11. Similar results were observed in multiple immune cells and immune functions, which were calculated using single-sample gene set enrichment analysis. The prediction of the efficacy of immunotherapy with TICA and TIDE databases proved that the lower SLC7A11 level has a better curative effect of anti-PD-1/PD-L1 and anti-CTLA4 treatment and less probability of immune escape and immune dysfunction. All the evidence mentioned above indicated that SLC7A11 was highly relevant to immune status in LUAD, and it may be involved in regulating immune cells and immune functions.

In the study, we proved that SLC7A11 was closely related to the prognosis and immune status of LUAD patients. However, there were still some limitations that needed to be addressed. (1) All data and participants were obtained from public databases: TCGA and GEO. Therefore, the potential selection bias could not be precluded entirely. (2) The study was retrospective, and prospective study validation was still needed. (3) All data used in the study were from microarray expression and RNA-seq. Therefore, it lacked basic experiments in vitro and in vivo for further verification, such as PCR and IHC. (4) Although we predicted the relative probabilities to respond to immunotherapy, no treatment-related clinical experiments were presented. Hence, clinical experiments for assessing the association between SLC7A11 and the efficacy of immunotherapy were requisite.

## 5. Conclusion

In summary, in the present study, we collected the expression profile of SLC7A11 from the TCGA and GEO databases and analyzed the differential expression level of SLC7A11 between normal and LUAD tissues. We found that the expression level of SLC7A11 in LUAD was significantly increased, which was an unfavorable factor for the prognosis. In addition, we identified several immune-related pathways enriched in the low-level SLC7A11 group using GSEA analysis, and SLC7A11 was highly correlated with the immune system and therapeutic effect of immunotherapy, showing that regulating the immune system may be one approach that SLC7A11 functions. In addition, SLC7A11 could be an effective method for evaluating LUAD prognosis and the therapeutic efficacy of immunotherapy, and targeting SLC7A11 could be a novel treatment mean for LUAD.

## Acknowledgments

We acknowledge the TCGA and GEO database for providing their platforms and contributors for uploading their meaningful datasets.

## Author contributions

**Formal analysis:** Xiaoyu Wu, Sheng Wang.

**Funding acquisition:** Sheng Wang.

**Methodology:** Xiaoyu Wu, Kaifang Chen.

**Project administration:** Kaifang Chen.

**Software:** Xiaoyu Wu.

**Writing – original draft:** Sheng Wang.

**Writing – review & editing:** Kaifang Chen.

## Supplementary Material


