# Supplementary figures and images for: Bulk RNA-seq and scRNA-seq reveal SLC7A11, a key regulatory molecule of ferroptosis, is a prognostic-related biomarker and highly related to the immune system in lung adenocarcinoma
Source: Medicine (Baltimore). 2023 Sep 15;102(37):e34876. doi: 10.1097/MD.0000000000034876 (PMC10508384; doi:10.1097/MD.0000000000034876)

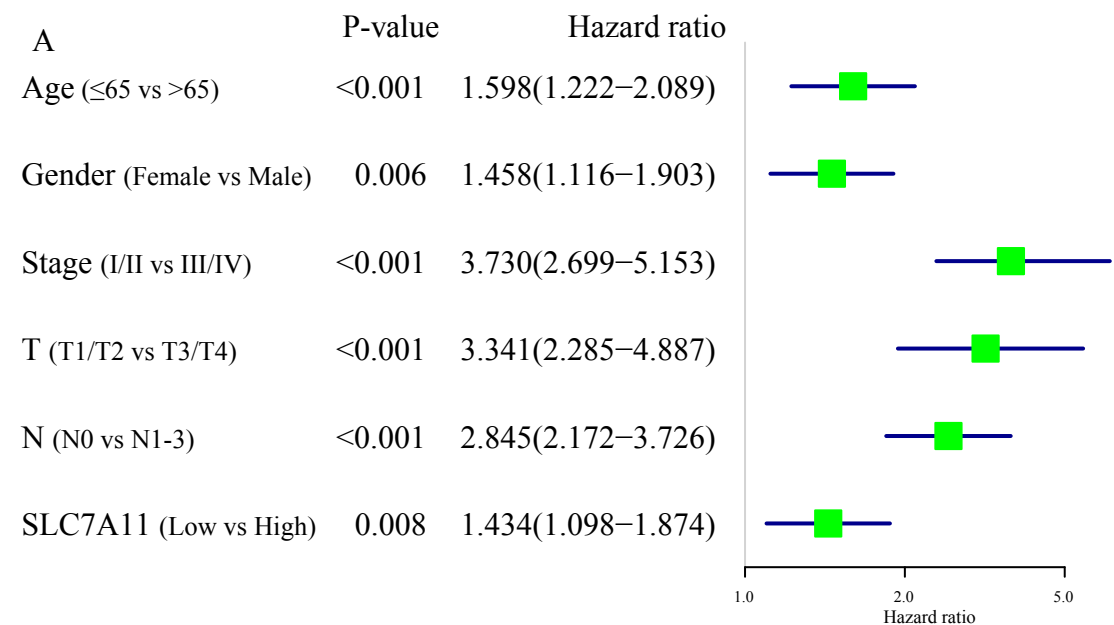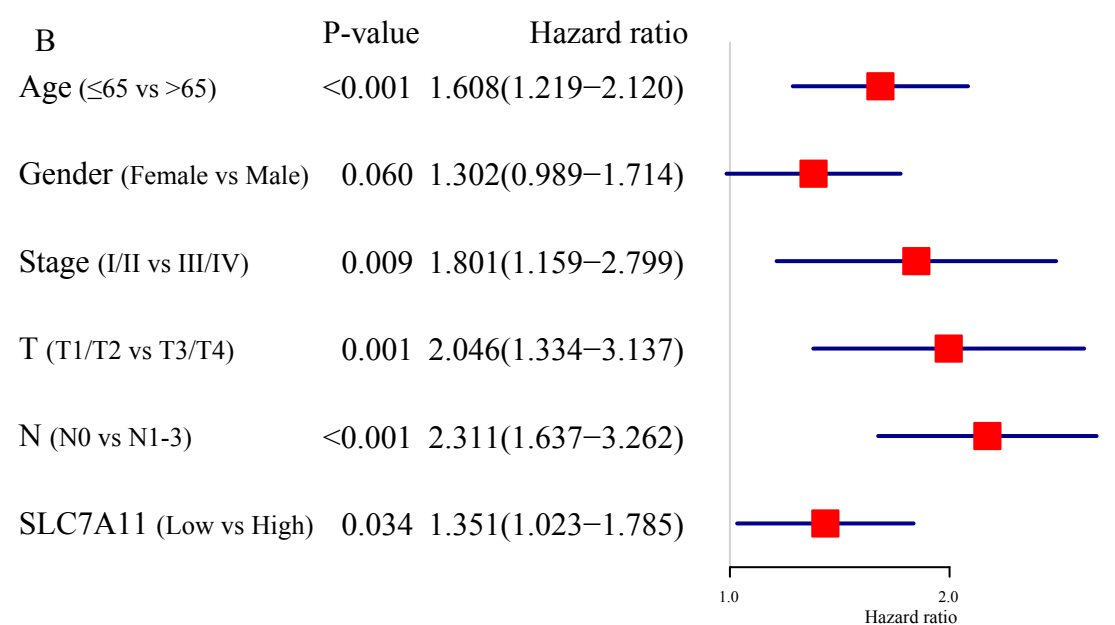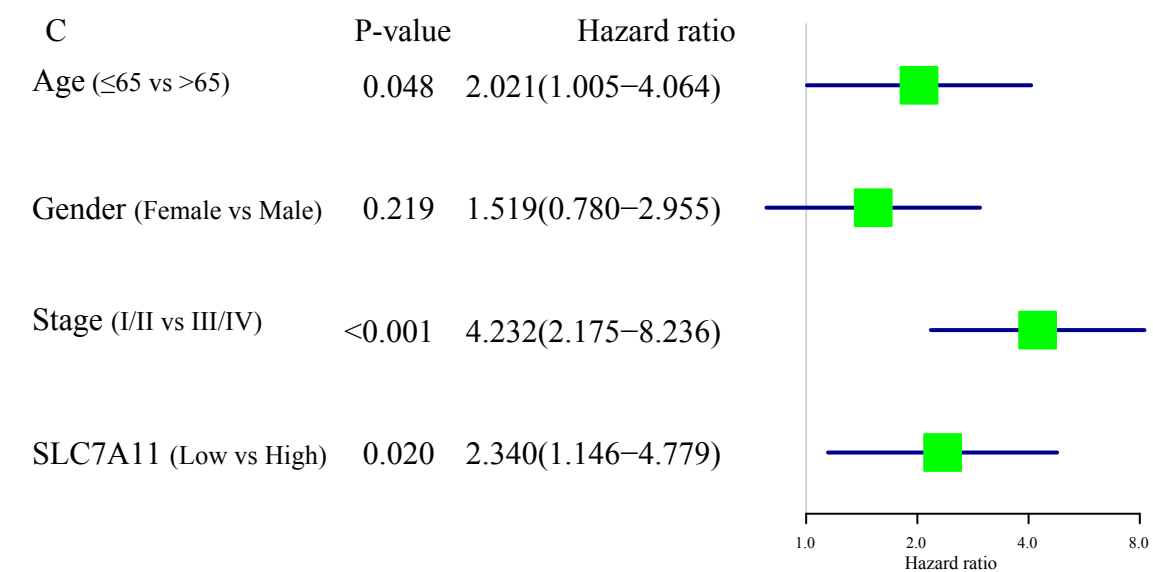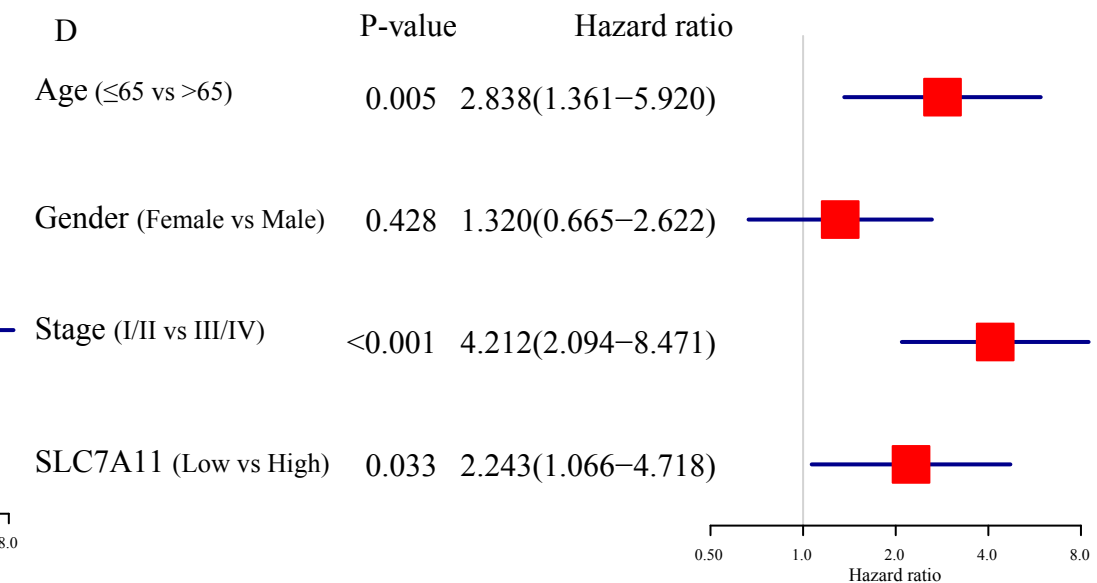

Supplement: Supplementary file 1 [file medi-102-e34876-s001.pdf]
